# Supplementary material for: The long non-coding RNA HOTAIR is transcriptionally activated by HOXA9 and is an independent prognostic marker in patients with malignant glioma
Source: Oncotarget. 2018 Feb 28;9(21):15740–56. doi: 10.18632/oncotarget.24597 (PMC5884661; doi:10.18632/oncotarget.24597)
Supplement: Supplementary file 1 [file oncotarget-09-15740-s001.pdf]

The long non-coding RNA *HOTAIR* is transcriptionally activated by HOXA9 and is an independent prognostic marker in patients with malignant glioma

SUPPLEMENTARY MATERIALS

Supplementary Table 1: Clinicopathological features of glioma patients from TCGA, Oncomine, REMBRANDT, and our datasets (Portuguese and French). See Supplementary_Table_1

Supplementary Table 2: Univariate (Log-rank) and multivariate (Cox proportional regression model) analysis of *HOTAIR* prognostic value in IDH-wt GBM patients from TCGA microarray data

TCGA – IDH-wt GBM – microarrays				
	n	Overall Survival		
		Median (95% CI)[a]	*p*-value (Log-rank)	*p*-value (Cox)
***HOTAIR* expression**				
Positive	126	370 (328.9–411.1)	**0.032**	**0.036**
Negative	241	447 (399.5–494.5)		
**Age**				
>75	44	187 (92.8–281.2)	**<0.0001**	**0.003**
≤75	323	442 (394.0–490.0)		
**Gender**				
Male	225	394 (355.2–432.8)	0.130	**0.023**
Female	142	453 (337.0–569.0)		
**KPS[b]**				
≥80	200	504 (441.2–566.8)	**<0.0001**	**<0.0001**
<80	79	323 (208.2–437.8)		
**Radio- & Chemotherapy**				
Yes	262	476 (432.9–519.1)	**<0.0001**	**<0.0001**
No	78	111 (65.7–156.3)		
**Additional Radio- & Chemotherapy**				
Yes	32	598 (400.9–795.1)	0.059	0.282
No	306	394 (354.4–433.6)		

n - number of patients; [a]Median survival with 95% confidence intervals, in days; [b]KPS was used as continuous variable for the Cox model.

Supplementary Table 3: Univariate (Log-rank) and multivariate (Cox proportional regression model) analysis of *HOTAIR* prognostic value in GBM patients from REMBRANDT dataset

REMBRANDT – GBM				
	n	**Overall survival**		
		**Median (95% CI)[a]**	***p*-value (Log-rank)**	***p*-value (Cox)**
***HOTAIR* expression**				
Positive	48	15.8 (12.8–18.8)	**0.005**	**0.034**
Negative	19	37.4 (11.9–62.9)		
**Age**				
>50	26	15.8 (9.8–21.8)	**<0.0001**	**0.002**
≤50	29	32.0 (18.5–45.5)		
**Gender**				
Male	16	27.5 (3.8–51.2)	0.379	0.275
Female	8	20.0 (14.5–25.5)		
**Institution**				
TJU	12	9 (3.9–14.1)	**0.001**	0.402
NIH NOB	10	26.6 (11.0–48.2)		
PITT	2	6.9 (N/A)		
UCSF	4	30.8 (N/A)		
HLMCC	5	19.0 (2.4–14.3)		
UCLA	2	27.5 (N/A)		
DFCI	1	5.4 (N/A)		
HFH	27	18.0 (13.8–22.2)		
MDACC	2	3.7 (N/A)		
JHH	2	18.0 (14.3–21.7)		

n - number of patients; TJU - Thomas Jefferson University; NIH NOB - National Institute of Health Neuro-Oncology Branch; PITT - University of Pittsburgh; UCSF - University of California, San Francisco; HLMCC - H. Lee Moffitt Cancer Center; UCLA - University of California, Los Angeles; DFCI - Dana-Farber Cancer Institute; HFH - Henry Ford Hospital; MDACC - M.D. Anderson Cancer Center; JHH - John Hopkins Hospital; [a]Median survival with 95% confidence intervals, in months.

Supplementary Table 4: Univariate (Log-rank) and multivariate (Cox proportional regression model) analysis of *HOTAIR* prognostic value in grade III (n = 28) glioma patients from the French cohort

French cohort							
		**Overall survival**					
		**Univariate analysis**			**Multivariate analysis[e]**		
	n	***p*-value (Log-rank)**	**HR**	**95% CI**	***p*-value (Cox)**	**HR**	**95% CI**
***HOTAIR* expression**	28	**0.002**	3.05e+12	32509–2.87e+20	**0.022**	1.04e+13	71.92–1.49e+24
**Age at diagnosis (years)**	28	**0.015**	1.04	1.00–1.08	0.974	0.999	0.94–1.06
**Gender[a]**	28	0.396	1.46	0.61–3.50	0.896	0.927	0.30–2.89
**KPS**	28	**<0.0001**	0.92	0.89–0.97	**0.001**	0.891	0.83–0.95
**Treatment[b]**	28	**<0.0001**	N/A	N/A	1.000	3.79e-16	N/A
**IDH status[c]**	28	**0.020**	0.21	0.05–0.93	0.646	0.572	0.05–6.19
**1p/19q codeletion[d]**	25	0.052	N/A	N/A	N/A	2.11e-20	N/A

n - number of patients; N/A – not applicable; [a]Female *versus* male; [b]non-treated *versus* treated; [c]non-mutated *versus* mutated; [d]1p19q non-codeleted *versus* codeleted; [e]Confusing factors used in the multivariate analysis: sex, age at diagnosis, KPS, treatment, and IDH and 1p/19q codeletion status. *HOTAIR* expression and age were used as continuous variable for the Cox model.

Supplementary Table 5: Univariate (Log-rank) and multivariate (Cox proportional regression model) analysis of *HOTAIR* prognostic value in grade II glioma patients from TCGA RNA-seq data

TCGA dataset – RNA-seq				
	n	**Overall survival**		
		**Median (95% CI)[a]**	***p*-value (Log-rank)**	***p*-value (Cox)**
***HOTAIR* expression[b]**				
Positive	29	94.5 (59.9–129.1)	**0.020**	**0.032**
Negative	197	130.8 (83.5–178.1)		
**Age[b,c]**				
>Median	134	95.6 (54.4–136.8)	0.210	**0.021**
≤Median	92	130.8 (77.8–183.8)		
**Gender**				
Male	125	117.4 (90.1–144.7)	0.503	0.621
Female	101	130.8 (82.6–179.0)		
**Molecular subtype**				
IDH-wt	12	25.5	**0.047**	0.348
IDH-mut	75	87.5 (41.5–133.5)		0.994
IDH-mut & 1p19q codel	48	154.4 (64.5–244.3)		0.567

n - number of patients; [a]Median survival with 95% confidence intervals (months); [b]*HOTAIR* expression and age were used as continuous variable for the Cox model; [c]Age median is 41.

Supplementary Table 6: Univariate (Log-rank) and multivariate (Cox proportional regression model) analysis of *HOTAIR* prognostic value in grade III glioma patients from TCGA RNA-seq data

		TCGA dataset – RNA-seq		
	n	**Overall survival**		
		**Median (95% CI)[a]**	***p*-value (Log-rank)**	***p*-value (Cox)**
***HOTAIR* expression[b]**				
Positive	89	26.8 (20.6–33.0)	**<0.0001**	0.395
Negative	151	75.0 (55.8–94.2)		
**Age[b,c]**				
>Median	132	34 (21.0–47.0)	**<0.0001**	**<0.0001**
≤Median	108	114.1 (52.7–175.5)		
**Gender**				
Male	138	67.5 (42.7–92.3)	0.433	0.541
Female	102	54.8 (32.2–77.4)		
**Molecular subtype**				
IDH-wt	43	18.4 (15.7–21.1)	**<0.0001**	**<0.0001**
IDH-mut	62	57.9 (32.3–83.6)		
IDH-mut & 1p19q codel	37	62 (39.6–84.4)		

n - number of patients; [a]Median survival with 95% confidence intervals (months); [b]*HOTAIR* expression and age were used as continuous variable for the Cox model; [c]Age median is 41.

Supplementary Table 7: Sequences of the primers used for RT-PCR and qPCR analysis

Gene	Sense sequence (5′-3′)	Antisense sequence (5′-3′)	Annealing T (° C)
HOTAIR (PD)	CAGTGGGGAACTCTGACTCG	GTGCCTGGTGCTCTCTTACC	60
HOTAIR (FD)	GGTAGAAAAAGCAACCACGAAGC	ACATAAACCTCTGTCTGTGAGTGCC	60
HOTAIR - ChIP	ATGGACGCTCTCGTTTGTTC	CGGGTGCAAGATAAACCACT	60
HOXA9 (PD)	GCCCGTGCAGCTTCCAGTCC	GAGCGCGCATGAAGCCAGTTG	61
HOXA9 (FD)	GCCGGCCTTATGGCATTAAA	AGGGACAAAGTGTGAGTGTCA	60
hGUS	CCTGTGACCTTTGTGAGCAA	GTGCCCGTAGTCGTGATACC	56
TBP	GAGCTGTGATGTGAAGTTTCC	TCTGGGTTTGATCATTCTGTAG	60
HPRT1	TGAGGATTTGGAAAGGGTGT	GAGCACACAGAGGGCTACAA	60
PPIA	TCTGGTTCCTTCTGCGTGAA	CACCCAGGGAATACGTAACCA	60
HOTAIR - MET	AGTAAAGCGTATAAGGGTTTAGTGC	CGACTACGACGCTACCTTACG	62–57 (−1º C/cycle)
HOTAIR - UNMET	AGTGTATAAGGGTTTAGTGTGGGG	CAACTACAACACTACCTTACACTCC	

PD - Portuguese dataset; FD - French dataset.

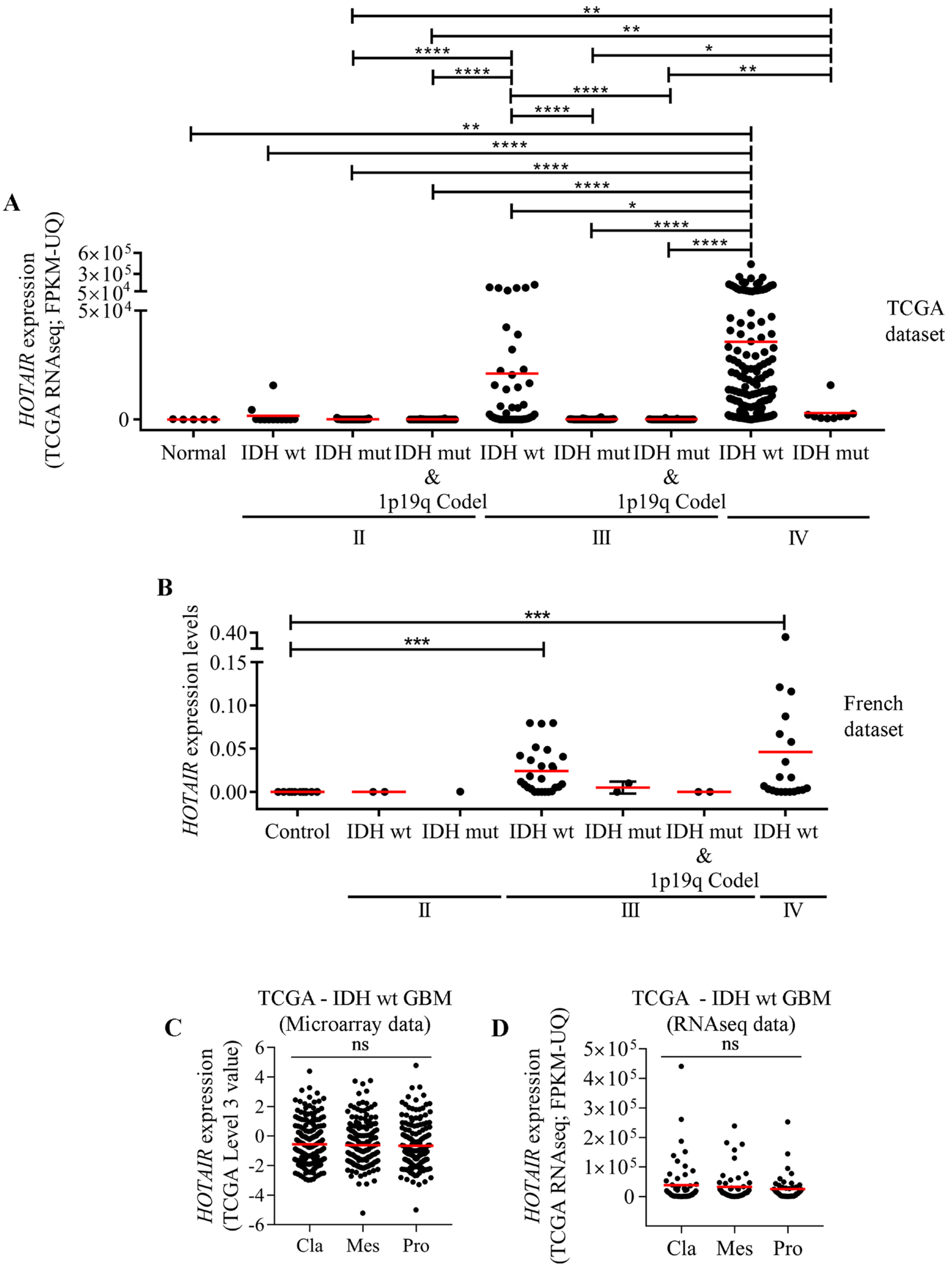

Supplementary Figure 1: *HOTAIR* is highly expressed in IDH-wt GBM and does not associate with molecular subtypes of GBM. (**A**) Expression levels of *HOTAIR* in 429 gliomas, stratified according to WHO grade, IDH and 1p/19q codeletion statuses (12 IDH-wt, 75 IDH-mut, and 48 IDH-mut and 1p/19q codeleted grade II gliomas; 43 IDH-wt, 62 IDH-mut, and 37 IDH-mut and 1p/19q codeleted grade III gliomas; 143 IDH-wt and 9 IDH-mut glioblastomas (GBM); and 5 unmatched normal brains from the TCGA RNA-seq data). *HOTAIR* is expressed (TCGA data "level 3" values ≥ 0) in 34.2 % (n = 126) of IDH-wt GBM samples and in 1 IDH-mut GBM (3%) and 1 IDH-wt grade II glioma (100%). (**B**) *HOTAIR* expression levels in 51 gliomas, stratified according to WHO grade, IDH and 1p/19q codeletion statuses (2 IDH-wt and 1 IDH-mut grade II gliomas; 24 IDH-wt, 2 IDH-mut and 2 IDH-mut and 1p/19q codeleted grade III gliomas; 20 IDH-wt GBM; and 10 normal brain samples from the French dataset). (**C–D**) Association between *HOTAIR* expression and the molecular subtypes of GBM in 524 IDH-wt GBMs analyzed by microarrays (C) and 158 IDH-wt GBMs analyzed by RNA-seq (D). *HOTAIR* does not associate with any of the molecular subtypes. Cla – classical; Mes – mesenchyma; Pro – proneural.

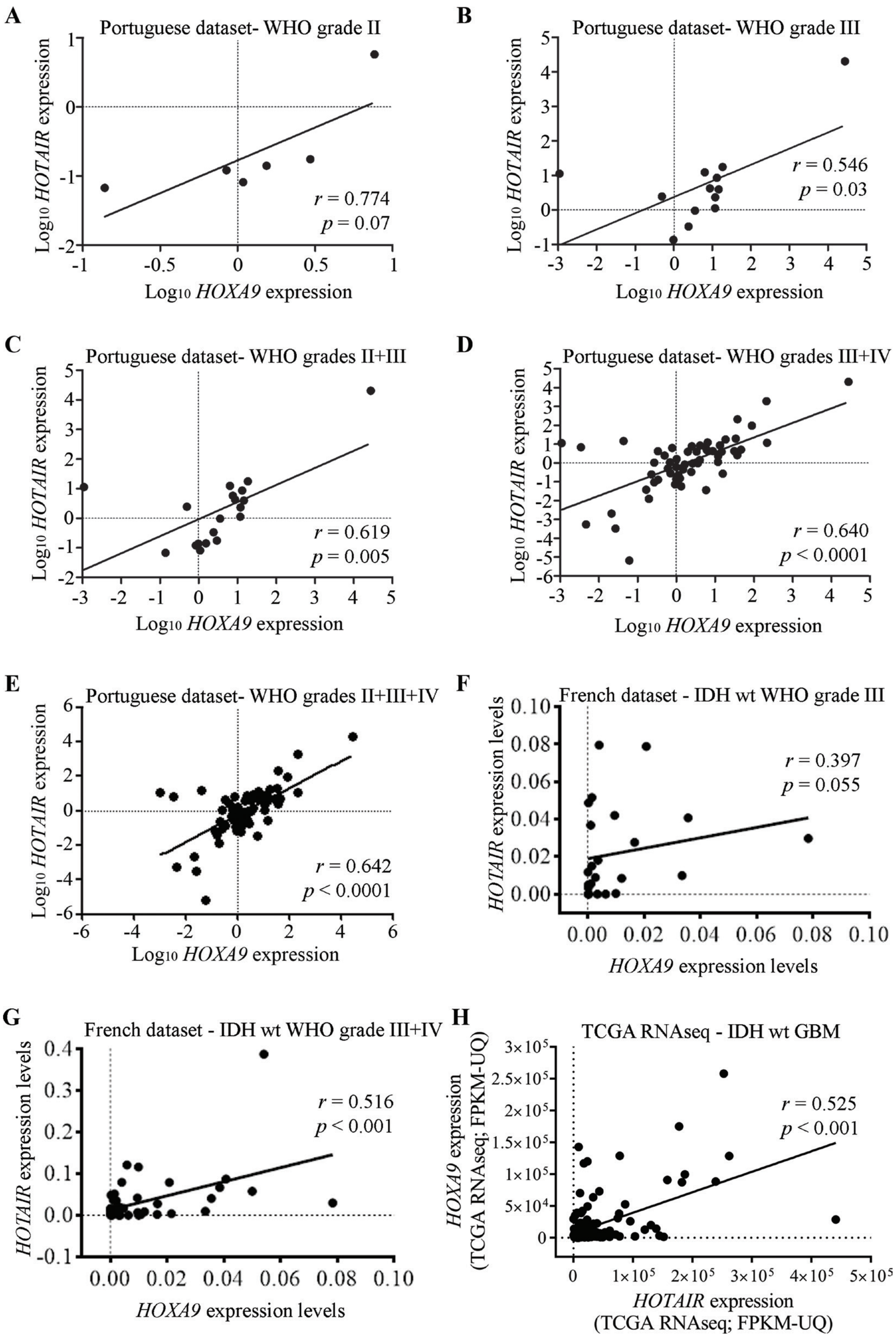

Supplementary Figure 2: Correlations between *HOTAIR* and *HOXA9* expression levels in clinical specimens of different glioma grades. (**A–E**) Correlation graphs of *HOTAIR* and *HOXA9* expression levels in (A) grade II, (B) grade III, (C) and in combinations of grade II and III, (D) grades III and IV, and (**E**) grades II, III and IV gliomas from the Portuguese dataset. (A) Pearson's $r = 0.774$, $p = 0.07$; (B) Pearson's $r = 0.546$, $p = 0.03$; (C) Pearson's $r = 0.619$, $p = 0.005$; (D) Pearson's $r = 0.640$, $p < 0.0001$; (E) Pearson's $r = 0.642$, $p < 0.0001$. (**F–G**) Correlation graphs of *HOTAIR* and *HOXA9* expression levels in (F) IDH-wt glioma grade III, and (**G**) combination of IDH-wt glioma grades III and IV from the French dataset. (F) Pearson's $r = 0.397$, $p < 0.055$; (G) Pearson's $r = 0.516$, $p < 0.001$. (**H**) Correlation graphs of *HOTAIR* and *HOXA9* expression levels collected by RNA-seq in IDH-wt GBM from the TCGA. Pearson's $r = 0.525$, $p < 0.0001$.

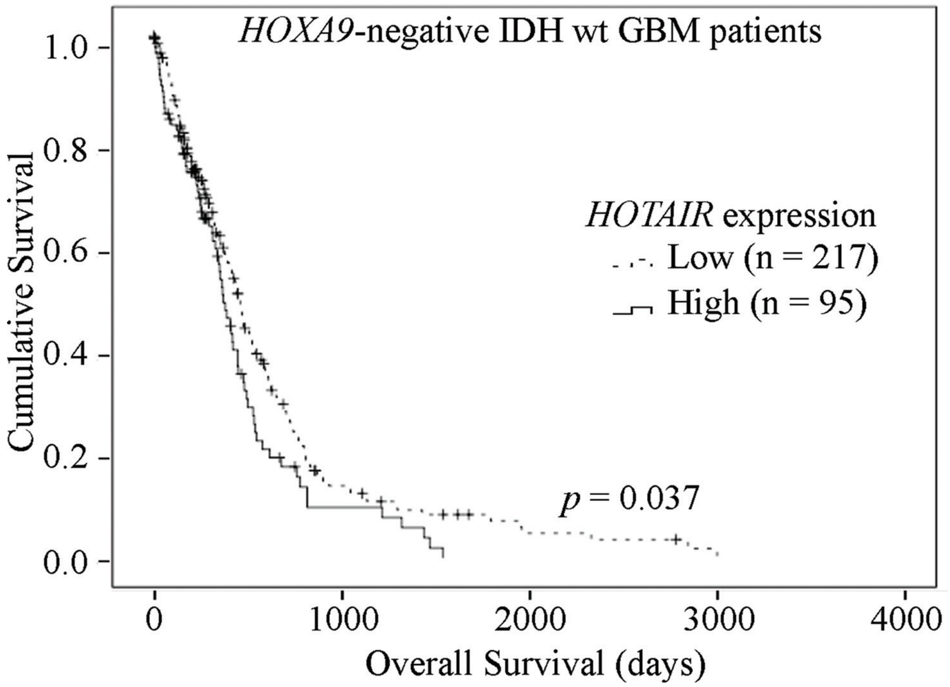

Supplementary Figure 3. Prognostic value of *HOTAIR* in IDH-wt GBM patients with low expression of *HOXA9*. Kaplan-Meier overall survival curves in a subset of 312 patients IDH-wt GBMs with low-*HOXA9* expression, stratified by *HOTAIR* levels (TCGA dataset). Within this subgroup of *HOXA9*-low IDH-wt GBMs, high expression of *HOTAIR* still identifies patients with a significantly shorter overall survival (Log-rank $p = 0.037$).

REFERENCES

1. Cancer Genome Atlas Research Network. Comprehensive genomic characterization defines human glioblastoma genes and core pathways. Nature. 2008; 455:1061–8. https://doi.org/10.1038/nature07385.
2. Murat A, Migliavacca E, Gorlia T, Lambiv WL, Shay T, Hamou MF, de Tribolet N, Regli L, Wick W, Kouwenhoven MC, Hainfellner JA, Heppner FL, Dietrich PY, et al. Stem cell-related “self-renewal” signature and high epidermal growth factor receptor expression associated with resistance to concomitant chemoradiotherapy in glioblastoma. J Clin Oncol. 2008; 26:3015–24. https://doi.org/10.1200/JCO.2007.15.7164.
3. Phillips HS, Kharbanda S, Chen R, Forrest WF, Soriano RH, Wu TD, Misra A, Nigro JM, Colman H, Soroceanu L, Williams PM, Modrusan Z, Feuerstein BG, et al. Molecular subclasses of high-grade glioma predict prognosis, delineate a pattern of disease progression, and resemble stages in neurogenesis. Cancer Cell. 2006; 9:157–73. https://doi.org/10.1016/j.ccr.2006.02.019.
4. Sun L, Hui AM, Su Q, Vortmeyer A, Kotliarov Y, Pastorino S, Passaniti A, Menon J, Walling J, Bailey R, Rosenblum M, Mikkelsen T, Fine HA. Neuronal and glioma-derived stem cell factor induces angiogenesis within the brain. Cancer Cell. 2006; 9:287–300. https://doi.org/10.1016/j.ccr.2006.03.003.
5. Freije WA, Castro-Vargas FE, Fang Z, Horvath S, Cloughesy T, Liau LM, Mischel PS, Nelson SF. Gene expression profiling of gliomas strongly predicts survival. Cancer Res. 2004; 64:6503–10. https://doi.org/10.1158/0008-5472.CAN-04-0452.
