# Supplementary material for: The long non-coding RNA HOTAIR is transcriptionally activated by HOXA9 and is an independent prognostic marker in patients with malignant glioma
Source: Oncotarget. 2018 Feb 28;9(21):15740–56. doi: 10.18632/oncotarget.24597 (PMC5884661; doi:10.18632/oncotarget.24597)
Supplement: Supplementary file 2 [file oncotarget-09-15740-s002.docx]

**Supplementary Table 1: Clinicopathological features of glioma patients from TCGA, Oncomine, REMBRANDT, and our datasets (Portuguese and French)**

| **Datasets** | | **Diagnosis** | **WHO grade** | **No. Cases** | **Age at diagnosis (median [IQR])** | **Male/female ratio** | ***HOTAIR*-Expression^a^ (%)** |
| --- | --- | --- | --- | --- | --- | --- | --- |
| **TCGA [1]** | **Microarray data** | IDH-wt | II | 1 | 43 [N/D] | N/D | 1 (100) |
|  |  | IDH-mut | II | 3 | 43 [N/D] | 2 | 0 (0) |
|  |  | IDH-mut & 1p19q codel | II | 3 | 42 [N/D] | 0.5 | 0 (0) |
|  |  | IDH-wt | III | 1 | 38 [N/D] | N/D | 0 (0) |
|  |  | IDH-mut | III | 12 | 36.5 [32.8-41.3] | 11 | 0 (0) |
|  |  | IDH-mut & 1p19q codel | III | 6 | 38.5 [33.3-49.8] | 1 | 0 (0) |
|  |  | IDH-wt | IV | 368 | 60.9 [52.3-69.4] | 1.6 | 126 (34.2) |
|  |  | IDH-mut | IV | 30 | 37.4 [28.4-45.6] | 2 | 1 (3.3) |
|  | **RNAseq data** | IDH-wt | II | 12 | 37.5 [26-51.5] | 2 | 4 (33.3) |
|  |  | IDH-mut | II | 75 | 36 [30-43] | 1.1 | 20 (26.7) |
|  |  | IDH-mut & 1p19q codel | II | 48 | 39 [31.8-53.3] | 1 | 9 (18.8) |
|  |  | IDH-wt | III | 43 | 58 [47.5-63] | 0.8 | 32 (74.4) |
|  |  | IDH-mut | III | 62 | 35.5 [31-41] | 2.4 | 20 (32.3) |
|  |  | IDH-mut & 1p19q codel | III | 37 | 51 [40-56] | 1.3 | 6 (16.2) |
|  |  | IDH-wt | IV | 143 | 62.7 [53.4-70.9] | 1.9 | 141 (98.6) |
|  |  | IDH-mut | IV | 9 | 34.7 [30.9-43.7] | 3.5 | 9 (100) |
| **Oncomine:** | **Murat [2]** | GBM | IV | 80 | 56.5 [50.5-61.0] | 0.73 | 2 (2.5) |
|  | **Phillips [3]** | AA | III | 24 | 35 [31.0-43.0] | 0.66 | 2 (8.3) |
|  |  | GBM | IV | 76 | 49 [40.8-55.0] | 0.68 | 7 (9.2) |
|  | **Sun [4]** | DA | II | 7 | N/A | N/A | 0 (0) |
|  |  | ODG | II | 38 | N/A | N/A | 0 (0) |
|  |  | AA | III | 19 | N/A | N/A | 3 (15.8) |
|  |  | ODG | III | 12 | N/A | N/A | 1 (8.3) |
|  |  | GBM | IV | 81 | N/A | N/A | 12 (14.8) |
|  | **Freije [5]** | AA | III | 8 | 34 [32.5-38.0] | 0.13 | 0 (0) |
|  |  | AOA | III | 7 | 32 [30.5-35.0] | 0.14 | 0 (0) |
|  |  | AODG | III | 11 | 35.5 [25.8-41.5] | 0.3 | 0 (0) |
|  |  | GBM | IV | 59 | 47 [39.0-60.5] | 0.44 | 1 (1.7) |
| **REMBRANDT** | | GBM | IV | 67 | 50 ± N/A | 0.79 | 48 (71.6) |
| **Portuguese dataset:** | **Coimbra** | DA | II | 3 | 38 [34.5-52.5] | 0.66 | 0 (0) |
|  |  | ODG | II | 1 | N/D | N/D | 0 (0) |
|  |  | AA | III | 2 | 28.5 [N/D] | 1 | 2 (100) |
|  |  | AOA | III | 3 | 66 [65.5-73.5] | 0.66 | 1 (33.3) |
|  |  | AODG | III | 1 | N/D | N/D | 1 (100) |
|  |  | GBM | IV | 27 | 66 [48.5-69.5] | 0.51 | 6 (22.2) |
|  |  | GSM | IV | 2 | 49 [N/D] | 1 | 0 (0) |
|  | **Braga** | DA | II | 1 | N/D | N/D | 0 (0) |
|  |  | GA | II | 1 | N/D | N/D | 1 (100) |
|  |  | AE | III | 2 | 66.5 [N/D] | 0.5 | 0 (0) |
|  |  | AGA | III | 1 | N/D | N/D | 0 (0) |
|  |  | AOA | III | 2 | 49.5 [N/D] | 0.5 | 1 (50) |
|  |  | AODG | III | 2 | 69 [N/D] | 1 | 0 (0) |
|  |  | ODG | III | 1 | N/D | N/D | 0 (0) |
|  |  | GBM | IV | 24 | 58.5 [48.8-67.3] | 0.33 | 7 (29.2) |
| **French dataset** | | IDH-mut | II | 2 | 51.3 [48.1-54.4] | 0 | 0 (0) |
|  |  | IDH-mut & 1p19q codel | II | 1 | N/D | N/D | 0 (0) |
|  |  | IDH-wt | III | 24 | 60 [48.9-70.4] | 0.5 | 12 (50) |
|  |  | IDH-mut | III | 2 | 37.4 [34.4-40.4] | 1 | 0 (0) |
|  |  | IDH-mut & 1p19q codel | III | 2 | 47 [46.9-47.1] | 0 | 0 (0) |
|  |  | IDH-wt | IV | 20 | 57.5 [54.3-64.8] | 0.66 | 9 (45) |
| AA - Anaplastic Astrocytoma; AE - Anaplastic Ependymoma; AGA - Anaplastic Gemisnocytic Astrocytoma; AOA - Anaplastic Oligoastrocytoma; AODG - Anaplastic Oligodendroglioma; DA - Diffuse Astrocytoma; GBM - Glioblastoma; GSM - Gliosarcoma; GA - Gemistocytic Astrocytoma; OAC - Oligoastrocytoma; ODG - Oligodendroglioma; N/A - not available; N/D - not defined; ^a^Samples were considered *HOTAIR*-high when microarray data values >0, or as *HOTAIR*-positive when RNAseq data were >0. | | | | | | | |
